# Hebrew-L2 speakers process auditory templatic words through their L1 processing mechanism with awareness of L2

**DOI:** 10.3389/fpsyg.2023.1164510

**Published:** 2023-06-06

**Authors:** Yael Laure, Sharon Armon-Lotem

**Affiliations:** ^1^Department of English Literature and Linguistics, Bar-Ilan University, Ramat Gan, Israel; ^2^The Multidisciplinary Brain Research Center, Bar-Ilan University, Ramat Gan, Israel

**Keywords:** word processing mechanism, sub-lexical morphemes, cross-linguistic influence, metalinguistic awareness, Hebrew-L2, rhyme judgment task

## Abstract

Bilingualism involves cross-linguistic influence (CLI) prompted by communicative function, which impacts the activation of the bilingual's L1/L2 language processing mechanisms. The current study examines the extent of CLI when semantic information is reduced. Semitic languages are known for their templatic words composed of intertwined sub-lexical root and template morphemes, entailing non-linear morphological processing. As the roots constitute the semantic core, comprehension was found to impact morphological processing among Hebrew-L2 readers. Herein, we assessed the processing mechanism activated among adult Hebrew-L2 bilinguals in an auditory rhyme judgment task that requires linear processing. The task was provided with Hebrew templatic word pairs comprising accentuated (meta)linguistic information irrespective of semantics: phonological co-occurrence restrictions (root), grammatical information of vocalic melodies (template), and contrastive stress. We hypothesized that CLI in Hebrew-L2 speakers would be reflected in low accuracy rates in rhyming pairs when linguistic information is accentuated, indicating distraction from the linear processing due to activation levels of the L2 processing mechanism caused by competing linguistic cues drawn on transferred linguistic information. We compared the performance of 58 adult Hebrew native speakers with 54 Hebrew-L2 speakers with Semitic and non-Semitic-L1. The findings demonstrate that Hebrew-L2 speakers performed the task using their L1 processing mechanism with varying activation levels of L2, showing awareness of the morphological processing due to the vocalic melody for non-Semitic-L1 and awareness of contrastive stress for Semitic-L1. The results confirm CLI also when semantics is reduced, elucidating how much CLI modulates the bilingual's language processing mechanism.

## 1. Introduction

Cross-linguistic influence (CLI) in bilinguals includes transfer of linguistic information between L1 and L2 and activation of these languages' processing mechanisms. The current study draws on the language mode hypothesis (Grosjean, 2001) and the unified competition model (MacWhinney, 2005) in a complementary manner, to explore the cross-linguistic influence of grammatical information when semantic information is reduced. Semitic languages are known for their templatic words composed of intertwined sub-lexical root and template morphemes, entailing language-specific non-linear processing. Examining activation of language mode in templatic word decomposition in an auditory rhyme judgment task, we probe CLI concerning metalinguistic information of the root and template and contrastive stress among Hebrew-L2 adult speakers, whose L1 is either Semitic or non-Semitic, compared to Hebrew native speakers. The influence was assessed in two-resolution levels: general awareness of the morphological processing and particular awareness of the root and template morphemes and stress. We hypothesized that CLI would occur when the linguistic information taps into induced or learned awareness but not subliminal awareness. We also hypothesized that the results would be affected by the bilingual's L1; therefore, we analyzed the results accordingly. To address our aims, we first present the unified competition model and the language mode hypotheses. Next, we explain the Hebrew templatic words and the sub-lexical root and template morphemes, and finally, we present the design of this study.

Bilingualism involves L1-L2 interplay in the bilingual's processing mechanism. The unified competition model (MacWhinney, 2005) premises that languages are used in the service of communicative function. Transfer of linguistic information between L1 and L2 increases the form-function matching possibilities, resulting in conflicting linguistic cues that compete to be selected by the language processing mechanism. The winning cue is the stronger and more reliable one, due to entrenchment obtained by neural circuits formed by (co)activation of the specific linguistic information. However, activation of the bilingual's languages is also affected by non-linguistic parameters, such as whether the bilingual is being spoken or listened to, the bilingual's proficiency (socioeconomic status, the usual mode of interaction), the situation (physical location, degree of formality), the form or content of the message (the topic, visual/aural modality, vocabulary), the function of the language act (communicating vs. creating social distance, taking part in an experiment), and research factors (aim, task used, organization, and type of stimuli) (Grosjean, 1998, 2001). The language mode hypothesis (Grosjean, 2001) holds that the bilingual's languages are activated in changeable levels at a given point in time, ranging from no activation to full activation, with one of the languages being the governing processing mechanism, which is also changeable. Thus, L2 can be either free of L1 interference or filtered through L1, or L1 and L2 change each other.

Studies among bilinguals with Hebrew-L2 have shown transfer of functional linguistic information. Influence of L2 on L1 has been demonstrated among Russian-Hebrew and Dutch-Hebrew children who applied non-native-like processing strategies of syntactic cues: case vs. word order (Janssen et al., 2015). Influence of L1 on L2 has been shown in lexical retrieval mechanism of object relative clauses among Russian-Hebrew children (Botwinik et al., 2016). Bidirectional transfer has been found in semantic word processing by sharing translation among English-Hebrew speakers who learned Hebrew as a first or second language (Degani et al., 2011). Moreover, although transfer is predicted to be minimized when language-specific properties exist in one of the bilingual's languages but not in the other (MacWhinney, 2005), morpho-syntactic features such as definiteness (only Hebrew), syntactic aspect (only Russian), and accusative case (both Russian and Hebrew) have been shown in bidirectional L1-L2 influence among Russian-Hebrew bilingual children (Meir et al., 2017).

Unlike children, who acquire L2 more completely than adults, adults need to coactivate L1-L2 linguistic knowledge by utilizing (meta)linguistic awareness, which is achieved by attention to the similarities and differences between the languages (MacWhinney, 2013; Bley-Vroman, 2018). The current study addresses CLI of language-specific metalinguistic information (apart from meaning) concerning the root and template and non-linear processing of templatic words among Hebrew-L2 adult bilinguals whose L1 is either Semitic or non-Semitic. According to the unified competition model, transfer of information or competition are not expected to occur because (i) the communicative function is not involved in this study, (ii) transfer is minimized when the linguistic information exists only in one of the bilingual's languages, as is the case with the non-Semiticn-L1, and (iii) Semitic languages share this particularity (McCarthy, 1981), rendering competition redundant, as is the case with the Semitic-L1. However, given that non-linguistic parameters also play a role and L1-L2 activation is dynamic, activation of the L2 processing mechanism, as evidence of linguistic transfer, may be seen due to the experiment requirements, modality, stimulus type, and (meta)linguistic awareness level. Therefore, these parameters should be handled and scrutinized with precision.

Semitic languages are known for their templatic words composed of intertwined two sub-lexical morphemes. The root, 2–6 (most common 3) consonantal phonemes, submitted to phonological co-occurrence restrictions, provide the semantic core. The template, vocalic pattern with or without fixed consonants, provide functional and grammatical information. Each morpheme may have more than one meaning. The word's meaning is a result of the joined morphemes and context. For example, in Hebrew, the words *xa*ʃ*av* (he thought/accountant) and *xi*ʃ*ev* (he calculated) are composed of the root *x*ʃ*v* (think/calculate) and the templates *-a-a-* and *-i-e-* (verbs in the past tense/*-a-a-* also denotes profession; historically, a different template), and *max*ʃ*ev* (computer) with the *ma—e-* template with fixed consonants (denoting a tool or a place). While the nominal system comprises about a hundred templates, the verbal system comprises seven templates of verbal structures called binyanim: in general, three for active voice (Pa'al, Pi'el, Hif'il), three for passive voice in mirror relations to the active ones (Nifa'l, Pu'al, Huf'al), and one mostly reflexive, reciprocal, and inchoative (Hitpa'el). The root-template derivational relations in the binyanim are relatively firm, though not without exceptions. An especially stringent active–passive derivational relation is the one of binyanim Pi'el-Pu'al, e.g., *xi*ʃ*ev-xu*ʃ*av* (calculated-was calculated). Derivations in Pa'al-Pu'al that share the same root may be semantically related (aka opaque) like *xa*ʃ*av-xu*ʃ*av* (thought-was calculated) or non-related like in *pasal-pusal* (canceled-was sculptured); however, they do not hold active–passive relations of the same action.

Composing words by intertwining the root and template sub-lexical morphemes entails non-linear processing, a Semitic linguistic particularity (aka the root-template mechanism). This contrasts with the universal approach that words are concatenative strings of phonemes. For example, the word *good* in English is a concatenative string of the phonemes /g/+/℧/+/*d*/, and *good*ness is a concatenative composition of the stem word *good* and the suffix -*ness*. Linear vs. non-linear processing of templatic words provokes an ongoing linguistic debate. Linguistic theories that advocate a stem-base lexicon deny the independent status of the root and template morphemes, providing linear processing mechanisms for Semitic templatic words (Aronoff, 1976; Bat-El, 1994, 2003; Benmamoun, 2003; Heath, 2003), so do computational theories, such as the Optimality Theory (Ussishkin, 2000, 2003). However, psycholinguistic research brings evidence for the independent representation of the root and template and non-linear processing, required for access to the lexicon and reading. Using masked priming paradigms experiment with lexical decision, repetition, and recognition tasks with words and non-words with real roots and with invented roots conditions, visual morphological priming effects were found among Hebrew native speakers in the verbal system (Deutsch and Frost, 2003) and in both verbal and nominal domains (Yablonski and Ben-Shachar, 2016), as real roots in non-words resulted in lower accuracy and longer response time compared to non-words with non-real roots or real words. Priming effects were also found in cross-modality models (visual and auditory), showing semantic-dependent priming effects in Arabic (Marslen-Wilson et al., 1994) or semantic-increasing effects in Hebrew (Gafni et al., 2019). Priming effects were also shown in the transposed root condition (phonemes in a different order) in visual experiments comparing Hebrew with English (Velan and Frost, 2007) or comparing Hebrew words of Semitic vs. non-Semitic origin (Velan and Frost, 2011), as well as in auditory repetition tasks (Oganyan et al., 2019).

Although these studies indicate awareness of Hebrew native speakers of the root and template morphemes, this awareness is semantic-dependent, as the studies used meaning-prompted experiments. The use of the printed word in Hebrew accentuates the root, tapping into the semantic core, as Hebrew is an abjad language. In addition, the use of printed words in a sentence requires semantics since the output is context-dependent. Moreover, the three-condition assessment consisting of real words, non-words with real roots, and non-words with non-real/illegal roots, as well as the transposed roots, in naming and lexical decision tasks compel semantic involvement, as the outcome taps into vocabulary knowledge. The question is, does non-linear processing occur when semantics is reduced, that is, due to (meta)linguistic information of the sub-lexical morphemes?

The linguistic impact of the root and template on non-linear processing of templatic words has been examined among Hebrew native speakers in a study using phonological awareness rhyme judgment task (saying if a pair of words rhymes) with auditory Hebrew templatic word stimuli with accentuated roots and templates for their linguistic information regardless of the meaning (Laure and Armon-Lotem, 2023a). This metalinguistic awareness measure, where semantics was reduced, creates an arena where the sub-lexical morphemes root and template compete with the syllables and sub-syllabic units for the processing mode: non-linear vs. linear, respectively. Success (accuracy) in this task indicates linear processing since the task requires parsing words linearly to syllables and phoneme discrimination. Hence, the low accuracy shown in rhyming pairs points to distraction from the linear processing. Low accuracy was shown in rhyming pairs where roots were identical in a pair, enabling the vocalic melody templates to stand out for their function, and when roots were transposed in a pair, minimizing the phonological feature realization in codas. The authors found the vocalic melody templates to have an abstract representation tapping into metalinguistic awareness of lexical-syntactic information and that transposed roots accentuate the application of the roots' phonological co-occurrence restrictions (Greenberg, 1950), tapping into subliminal linguistic knowledge of the computational phonological system.

A follow-up study comparing Hebrew native speakers with non-Hebrew speakers corroborated different processing mechanisms for Hebrew and non-Semitic non-Hebrew speakers (Laure and Armon-Lotem, 2023b). The non-Hebrew speakers processed rhyming and non-rhyming CVCVC pairs equally according to sub-syllabic units and phoneme similarity hierarchy in the final syllable, i.e., accuracy in recognizing rhymes decreases with the increase in similar phonemes in the final syllable unless the final syllables are identical (Lenel and Cantor, 1981). No awareness was shown among non-Hebrew speakers of the phonological co-occurrence restrictions or the abstract representation of the vocalic melody. Notably, the non-native Hebrew speakers scored low in non-rhyming identical templatic word pairs with contrastive stress (e.g., ***be****rex-be****rex*** (knee-blessed); stressed syllable in bold), likely because the contrastive stress is difficult to be perceived by speakers of languages with non-contrastive stress, like French (Segal and Kishon-Rabin, 2019). These results indicate that the grammatical information of the vocalic melody and the phonological co-occurrence restrictions are part of the (un)conscious metalinguistic knowledge of the Hebrew speaker. Having demonstrated that the root and template impact non-linear processing regardless of semantics among Hebrew native speakers, the question is, does this linguistic L2 particularity transfer and impact Hebrew-L2 bilinguals?

The Hebrew sub-lexical morphological non-linear processing has been examined among Hebrew-L2 readers in reading experiments that included words and non-words manipulated by four combinations of different/similar roots and patterns, reflecting on cross-linguistic influence (Norman et al., 2016, 2017). A study involving Hebrew-L1 and proficient Hebrew-L2 readers has found that morphological processing preceded lexical access for both Hebrew-L1 and proficient Hebrew-L2 readers from Indo-European and Semitic-L1 backgrounds, evident by processing strategies tuned to the root and template morphological processing in reading tasks (Norman et al., 2017). By contrast, a study including Hebrew-L2 learners in the early stages of learning has shown that the participants were modulated by L1 morphological background: L1-Indo-European beginning learners demonstrated sensitivity to the word pattern and word edges but not to the roots, and L1-Semitic beginning learners showed sensitivity to the fact that the word is the ensemble of both morphemes, as in Arabic, but not additive sensitivity to the root or template (Norman et al., 2016). However, although the morphological processing stands out in these experiments, it is impossible to disconnect the cognitive requirement associated with the written word in Hebrew, where the roots are salient, as words are written without vowels (abjad). Therefore, the question is, does cross-linguistic influence occur concerning the linguistic information associated with Hebrew templatic words regardless of semantics among Hebrew-L2 speakers, whose L1 is either Semitic or non-Semitic?

The present study explores this question using an auditory rhyming judgment task provided with Hebrew templatic words and comparing the performance of Hebrew-L2 speakers with Hebrew native speakers. Specifically, we examine the strength of L2 (meta)linguistic information concerning the morphological processing in general and the sub-lexical morphemes root and template and contrastive stress in particular by assessing activation of language modes in decomposing different templatic word stimulus types. The rationale for this study relies on the idea that the processing mechanism used by the bilinguals to decompose templatic words can reflect on the competition between conflicting linguistic information of sub-lexical units, i.e., root and template morphemes vs. syllables and sub-syllabic units. The auditory rhyme judgment task is utilized to designate the language modes' activation state (governing mechanism) and levels, while the stimulus types specify the sort of the transferred linguistic information of the root and template and stress.

Rhyme judgment tasks are part of a battery of tests that assess phonological awareness—the ability to understand that words are a series of sounds apart from their meaning. Phonological awareness is a language-universal construct (Branum-Martin et al., 2015) and has been shown to contribute to L2 consolidation (Zion et al., 2019). Recognizing rhymes requires identifying identical final words' vowel and consonant phonemes, which is successfully performed when parsing the words linearly into syllables, sub-syllabic units, and phonemes (Lewkowicz, 1980). Thus, the task compels linear processing, even more so when the stimuli are auditory since phonemes in spoken words are heard sequentially. Utilizing this task with templatic word stimuli creates an environment for competition between syllabic and morphemic sub-lexical units. The competition is even more difficult since the task requires mechanical decoding irrespective of semantics, while the roots get linguistic strength from the meaning. Moreover, non-linear processing is not beneficial in this task, as disentangling two intertwined sub-lexical morphemes in each word in a pair and then discriminating and comparing phonemes in two different sub-lexical units is cumbersome, costlier, and prone to errors. The strength of the syllabic units is granted not only by the task requirements but also by the universal phonological awareness construct. In addition, linear processing is also easier than non-linear processing (Upasana et al., 2022) and entrenched, as ceiling effects in this task have been seen by age six (Fox and Routh, 1975; Lewkowicz, 1980).

Since morphological processing occurs before lexical access (Norman et al., 2017), we hypothesized that despite the non-communicative function, the sub-lexical units (syllables/sub-syllabic units vs. morphemes) would compete due to the mechanical processing function required by the experiment. Success in this task, measured in accuracy, is indicative of linear processing. Low accuracy in non-rhyming pairs may occur due to inaccurate phoneme discrimination or to rhyme perception, i.e., phonemes are discerned but not always considered rhyme breakers. However, low accuracy in rhyming pairs is not expected in adults. Therefore, we considered low accuracy in this experiment as a distraction from linear processing. Given the balance of power of the two kinds of sub-lexical units, low accuracy in rhyming templatic word pairs would manifest cross-linguistic influence, indicating the strength of the linguistic L2 language-specific particularity of the sub-lexical root and template morphemes. Comparing the performance of Hebrew-L2 with Hebrew native speakers, we used two-resolution levels to assess cross-linguistic influence. The first aimed to capture a general awareness of the morphological processing of L2 Hebrew templatic words by examining accuracy in rhyming vs. non-rhyming pairs between and within stimulus types. The second focused on the linguistic information examined of each sub-lexical morpheme: phonological co-occurrence restrictions in transposed roots and the grammatical function of the vocalic melody in the template, both in rhyming pairs, and the contrastive stress in non-rhyming, non-stress-matched pairs.

Stimuli were sorted with linguistic precision to tap into the functional linguistic information irrespective of semantics. To avoid balanced-out results, we focused on one template type. All pairs were structure-matched with real roots. Manipulations emphasizing the root, template, and stress were achieved by combinations of different/identical real templates and different/identical real roots [+/-root,+/-template] in pairs, including transposed roots, vocalic melody templates, some of which violate binyanim relations, and non-stress-matched pairs. CLI was expected based on the (meta)awareness level, i.e., subliminal or induced or conscious. Specifically, we mainly expected salience of L2 linguistic information concerning the vocalic melody templates since the derivational function of vocalic melody is known, albeit to a lesser degree, also in non-Semitic languages (e.g., in English: choose-chose, begin-began-begun, etc.). In addition, the function of the template is prominent in the verbal system and taught, albeit on a semantic basis, in formal education, including Hebrew-L2 schools. Furthermore, it can be induced by usage. By contrast, awareness of the phonological co-occurrence restrictions was not predicted, as they are part of the subliminal computational system (regardless of whether submitted to grammar or statistical learning) (Berent, 2017). Awareness of the contrastive stress was partially expected. Unlike phonological awareness, stress is a language-specific construct (Branum-Martin et al., 2015). Acquisition of a second language facilitates awareness of stress when stress distinguishes between word meanings (Segal and Kishon-Rabin, 2019). However, the meaning of the words does not play a role in this task, which might impact the activation of L2. In addition, unlike Spanish, for example, where the contrastive stress is seen in the written modality, contrastive stress in Hebrew appears only in the oral/aural modality (it has no realization in Hebrew in the visual and written modality), and it can be resolved in context. Thus, the opportunities to induce or learn this awareness are reduced and modality-dependent.

Generally, we expected that cross-linguistic influence would be seen in the Hebrew-L2 speakers not just by the levels of (meta)linguistic awareness pronounced in the two-resolution levels but also by the level of similarity/difference between L1 and L2. Forces based on L1-L2 similar/different mechanisms and linguistic information are not equal when the Hebrew-L2 bilinguals have Semitic-L1 or non-Semitic-L1. It is difficult to tell which language mechanism is activated when L1 is Semitic, due to shared characteristics, and when L1 is non-Semitic, as linguistic transfer is expected to be minimized when the linguistic information pertains to only one of the bilingual's languages (MacWhinney, 2005). Therefore, precision is required. Although templatic words, root and template, and non-linear processing are common in Semitic languages (McCarthy, 1981), they have different manifestations. For example, the active–passive vocalic melody templates differ in length: The vocalic melody in the verbal system in Arabic includes three vowels [*-a-a-a* (active) *-u-i-a* (passive)], as opposed to two vowels [*-i-e-* (active) *-u-a-* (passive)] in Hebrew. In addition, beginner Hebrew-L2 learners with Semitic-L1 did not show sensitivity to the root or template in reading (Norman et al., 2016). In addition, as mentioned above, functional vocalic melody pattern is not exclusive to Semitic languages, although much more pervasive. Also, contrastive stress is language-specific, regardless of language family affiliation: contrastive stress in Hebrew, Spanish, and English vs. non-contrastive in French and Arabic (Segal and Kishon-Rabin, 2019). Therefore, we addressed the results using a multi-layer analysis: one of the Hebrew-L2 as a whole and the other separated by the bilinguals' L1.

Altogether, the task chosen, the precision in stimulus types, the two-resolution evaluation, and the multi-layer analysis enable the examination of cross-linguistic influence, including language activation and transfer of (meta)linguistic information.

## 2. Materials and methods

### 2.1. Participants

A total of 186 adults participated online; all declared not to have hearing problems. Participants who responded to < 85% of the stimuli were removed from the sample. 112 participants finished the experiment. 58 were Hebrew native speakers (Heb1) (ages 20–82 years, 39 female speakers), and 54 were Hebrew-L2 speakers (Heb2) (ages 21–82, 38 female speakers), with L1 including a Semitic language (Arabic) and non-Semitic languages. The participants filled in a questionnaire regarding demographic details, education in categories matching worldwide distinctions (some school, high school diploma, some college, undergraduate, graduate, and postgraduate), linguistic background information about the age of acquiring Hebrew, the number of years they use it, and their level of Hebrew in speech, reading, and writing on a 0–10 self-rating scale (Table 1). Speech level was important due to the auditory modality in this experiment. Since the experiment was performed online, the Hebrew native speakers were asked to self-rate their Hebrew level on a 0–10 scale in speech, reading, and writing, to verify that participants who defined Hebrew as their mother tongue could be defined as L1 Hebrew speakers (Table 1). The study was approved by the ethics committee of the Faculty of Humanities, Bar-Ilan University.

**Table 1:** Participants' background characteristics—means (SD) by language group.

	**Hebrew native**		**Semitic-L1 Hebrew-L2** ^*^		**Non-Semitic-L1 Hebrew-L2** ^**^		**Hebrew-L2**	
**Categorical variables**	***n***	**%**	***n***	**%**	***n***	**%**	***n***	**%**
Number and Gender	58		16		38		54	
Female	39	67.2%	15	93.8%	23	60.5%	38	70.4%
Male	19	32.8%	1	6.35%	14	36.8%	15	27.8%
Non-binary					1	2.6%	1	1.9%
Education								
Non-Academic	11	19%	3	18.8%	3	7.9%	6	11.1%
Academic	27	46.5%	13	81.2%	29	76.3%	42	77.8%
Non-specific	20	34.5%	–	–	6	15.8%	6	11.1%
**Continuous variables**	***Mean***	***(SD)***	***Mean***	***(SD)***	***Mean***	***(SD)***	***Mean***	***(SD)***
Age in years	41.83 (20–82)	*(14.61)*	26.19 (21–32)	*(3.33)*	49.66 (23–82)	*(19.48)*	42.70 (21–82)	*(18.94)*
Acquired Hebrew at the age of	N/A		6.81)	*(3.67)*	17.71	*(10.72)*	14.48	*(10.45)*
Number of years Hebrew is used	N/A		11.19	*(8.53)*	17.55	*(15.53)*	15.67	*(14.06)*
Hebrew speaking level	9.78	*(0.53)*	7.13	*(2.03)*	6.05	*(2.60)*	6.37	*(2.48)*
Hebrew reading level	9.79	(0.67)	7.56	*(2.07)*	5.95	*(3.07)*	6.43	*(2.89)*
Hebrew writing level	9.69	(0.68)	6.94	*(2.52)*	5.32	*(3.14)*	5.80	*3.04)*

^*^Arabic.

^**^By alphabetic order: Berber, Dutch, English, Farsi, French, German, Hungarian, Russian, and Spanish.

### 2.2. Stimuli

The experiment included 205 stimulus pairs, comprising 64 rhyming (R) and 141 non-rhyming (NR) auditorily structure-matched pairs. All words (some archaic) were examined to be valid using the Even-Shoshan dictionary (Even-Shoshan, 1974) and The Academy of the Hebrew Language site (https://hebrew-academy.org.il/. Accessed October 15, 2022). All bi-syllabic words are affix-free templatic words of full 3-consonantal roots assessed by the phonemic representation, without weak roots (where one of the root consonants is missing/not transparent) or geminate roots (e.g., tss, grr, etc.). We excluded the phonemes /ʔ/ (א), /ʕ/ (ע), /h/ (ה) since they may alter the auditory syllable structure (e.g., CVCV בנה (*bana*) or VCVC ענד (*anad*) or CV.VC דאג (*da'ag*) instead of CVCVC). We also excluded words with suffix-like final -VCs, such as /-*im*/, /-*ot*/ (plural morphemes), /-*on*/, /-*it*/ (diminutive), and /*-an*/ (personality characteristics/profession in Hebrew, and Accusative case in Arabic). Frequency of the words in the language was not considered as the experiment is metalinguistic awareness oriented. No impact of frequency was predicted for word decomposition as the target is technical parsing to isolate and compare the rime (-VC) and phonemes of the words in a pair.

The stimuli encompass mono-syllabic CVC pairs, used as control, and bi-syllabic structure-matched pairs of templatic words composed of roots and templates, including pairs with templatic fixed consonants mVCCVC and CVCVC pairs of [+/–CR,+/–VM] [CR for consonantal root; VM for vocalic melody (template)] combinations in pairs, including transposed roots, templates violating binyanim relations, and non-stress-matched pairs. The purpose of each stimuli type is detailed below (see Table 2 for examples).

1. CVC (62) pairs (15R/47NR), representing all identical and contrasting coda possibilities. We use them to ensure phoneme discrimination and the ability to recognize rhymes. This group is also a basis for comparison of R/NR processing in mono-syllabic vs. bi-syllabic pairs. These pairs are not templatic words.
2. mVCCVC (21) pairs (7R/14NR), nominal templates with initial consonants indicating tools or place. This type is used for comparison of R/NR processing with the CVCVC template, to pinpoint similarity or differences between two types of templatic words. Similarity between this type and the Baseline in CVCVC enables emphasizing the accentuated roles of the root and the template in the CVCVC pairs.

**Table 2:** Examples of stimulus types.

		**R**		**NR**	
		**Example**	**Translation**	**Example**	**Translation**
1	CVC	*dag-xag*	fish-holiday	*sal-saʃ*	basket-threshold
2	mVCCVC	*mavreg-mazleg*	screwdriver-fork	*mivcar-migdal*	fortress-tower
3	Baseline[–CR,–VM](B(−))	*ʃimer-ʃalax*	preserved-sent	*ʃiger-sagar*	launched-closed
4	Baseline[–CR,+VM](B(+))	*mazal-kaval*	luck-complained	*gamad-?amat*	dwarf-dropped
5	TCR[–CR,+VM]				
	C132	–	–	*xatar-xarat*	rowed-engraved
	C321	–	–	*karas-sakar*	collapsed-surveyed
	C231	–	–	*zaram-ramaz*	flowed-hinted
	C312	–	–	*ʃitek-kiʃet*	paralyzed-decorated
	C213	*kalax-lakax*	flowed-took	–	–
6	HVM[+CR, –VM]	*dabur-dibur*	hornet-speech	*natav-nituv*	router-routing
	Binyanim Relations				
	MIX	*kaʃer-koʃer*	kosher-ties(verb)	–	–
	Pa'al -Pi'el	*lomed-limed*	learns-taught	–	–
	Pa'al-Pu'al	*saxak-suxak*	laughed-was played	–	–
	Semantic relatedness				
	Non-related	*bocer-bicer*	picked grapes-fortified	–	–
	Related	*ʃamen-ʃimen*	fat (adj)-greased	–	–
7	Stress[+CR,+VM]	–	–	*corex-**co**rex*	consumes-need
8	Stress[+CR, –VM]	–	–	*dover-**de**ver*	spokesman-plague

Trochaic stress in bold, otherwise iambic.

CR, consonantal root; HVM, highlighted-vocalic melody; NR, non-rhyming pairs; R, rhyming pairs; TCR, transposed-consonantal root; VM, vocalic melody.

Six combinations of the 122 CVCVC pairs.

3. *Baseline[–CR,–VM] (B(*−*))*. 24 pairs (10R/14NR) with different CRs (ranging 0–2 out of 3) and VMs (ranging 0–1 out of 2) within the pair's words; used for setting the baseline for CVCVC pairs for representing phoneme variety and comparison of R/NR processing.
4. *Baseline[–CR*,+*VM] (B(*+*))*. 14 pairs (8R/6NR) with different CRs (ranging 0–2 out of 3) and identical VMs within the pair's words; used for comparisons of R/NR processing and for comparison with the transposed root pairs to establish the phonological impact of the transposed phonemes.
5. *Transposed-CR[–CR*,+*VM] (TCR)*. 40 pairs (8R/32NR) with roots sharing the same phonemes in different positions (transposed roots) and identical VMs. The transposed root stimulus type highlights the phonological co-occurrence restrictions in the roots. The non-rhyming pairs are four times more the number the rhyme pairs since 3-consonantal roots have five swaps (named after the second word's alternation), four of which are non-rhyming pairs and one rhyming pairs (see Table 2 for swap examples). This stimulus type is used for comparison of R/NR processing with the Baseline(+) to evaluate the susceptibility of transposed roots to phonological restrictions compared to varying consonantal roots.
6. *Highlighted-VM[*+*CR*,−*VM] (HVM)*. 32 pairs (20R/ 12NR) with identical CRs and different VMs; used for comparison of R/NR processing, and also to examine in rhyming pairs the effect of binyanim relations: Pa'al-Pi'el, Pa'al-Pu'al, and MIX (no binyanim relations), as well as the impact of semantics by comparing pairs with vs. without semantic relatedness between the words in a pair (see Table 2 for examples).
7. *Stress[*+*CR*,+*VM] (Stress)*. 6 identical CRs and VMs in non-stress-matched (trochaic (in bold) vs. iambic) pairs, therefore non-rhyming pairs; used to examine awareness of the contrastive stress and its impact on processing in this task.
8. *Stress[*+*CR*,+*VM] (Stress(*−*))*. 6 identical CRs and different VMs in non-stress-matched (trochaic (in bold) vs. iambic) pairs, therefore non-rhyming pairs; used to examine the extent of the impact and awareness of the stress vis-à-vis the template.

The stimuli were recorded using the Audacity software in a feminine voice in a professional studio or a quiet room. All pairs started after 55 ms with 300 ms gap between the words in each pair. Following pre-trial pilot feedback, the pairs slightly varied in volume to keep participants alerted and focused on the task, and response time was limited to 2 seconds to avoid an unintuitive decision. The pairs were randomly divided into five sections, containing all stimulus types, and fully randomized within sections. The randomized order was similar for all participants.

### 2.3. Procedure

We spread the online experiment with information about the research's aim, requirements, and instructions available in eleven languages (by alphabetic order: Arabic, Chinese, English, Filipino, French, German, Hebrew, Italian, Portuguese, Spanish, and Russian) through emails and social media. The experiment was limited to computers only to increase uniformity in testing conditions. We added a hearing test to verify that the speakers of the participants' computers work. Participants filled in a questionnaire relating to demographic information and linguistic background, read the instructions about the task in which they were asked to follow their intuition, and performed a practice trial (as many times as they wanted) to familiarize themselves with the procedure and technical aspects of the real trial; no feedback was given to the participants in order not to impact or interfere in their rhyme judgment. Then, they started the experiment. The pairs were played sequentially, after a response was issued or 2 secs passed (displayed on a diminishing bar). The question “Does it rhyme?” and the Yes and No buttons constantly appeared on the screen in each section. Between sections, the participants watched a silent 15-sec nature video, which differed between sections but appeared in a fixed order. Moving from one section to another required pressing “continue” and “start” buttons; thus, the pause length between sections was the participant's choice. The participants' answers were recorded in the database in their raw values: Yes, No. Then, the answers were converted to Correct (1) or Error (0) according to the following criterion: If both words of a pair have stress-matched identical final syllable's vowel and coda (–VC), the pair is a rhyming pair; otherwise, it is non-rhyming.

### 2.4. Statistical analysis

Since it was an online experiment of binary answers, to rule out malicious participants (pressing Yes/No blindly), we calculated the probability of each participant blindly answering the experiment question “Does it rhyme?.” A participant that blindly chooses Yes (*y*) or No (*n*) has an empirical probability (*p*) *p*_*y*_ and *p*_*n*_, respectively. Similarly, the probability of a pair in question (*q*) being a rhyme is *p*_*n*_·*q*_*n*_+*p*_*y*_·*q*_*y*_ = *p*. We have *m* questions, and let the number of answers the participant answered correctly be *k*. With these parameters, we calculated the probability (or likelihood) of said person to answer *k* correct “random guesses” out of *m* questions using the binomial distribution formula: $\left(\begin{array}{c} m\\ k \end{array}\right)p^{k}{\left(1-p\right)}^{m-k}$. The results indicate that each subject has a probability of <0.05 (range from 0.051329145 to 2.7312E-48) of achieving their accuracy (see Table A1 in the Supplementary Material for more details). Hence, none of the results nearly 50% indicate by chance accuracy.

Correlations between the experiment's five sections (*r* range 0.716–0.926) indicate high stability and consistency; therefore, we analyzed the results without separating sections. We used the multilevel modeling (MLM) for repeated measures designs as it allowed us flexibility in modeling a more appropriate variance-covariance matrix, relative to the repeated measure ANOVA, and handling missing data using the full information maximum likelihood, performed using SPSS IBM V.27. In case of significant main effects or interaction effects, a further set of *post hoc* comparisons were performed. To avoid alpha inflation, a Bonferroni adjustment was applied.

## 3. Results

The results were analyzed *via* stimulus multi-layer analyses from two perspectives: comparison of Hebrew native speakers (Heb1) with the entire sample of Hebrew-L2 speakers (Heb2) and comparison of Hebrew native speakers with Hebrew-L2 speakers discerned by the participants' mother tongue, Semitic (S-Heb2) vs. non-Semitic (nS-Heb2), to explore the source of the cross-linguistic influence.

### 3.1. Processing rhyming vs. non-rhyming pairs

####  3.1.1. Mono-syllabic CVC vs. Bi-syllabic CVCVC pairs

To examine whether Heb1 and Heb2 process similarly rhyming and non-rhyming pairs in mono-syllabic CVC and bi-syllabic CVCVC stimuli, we compared accuracy according to length by rhyme value (CVC-NR/CVC-R/CVCVC-NR/CVCVC-R) × language group (Heb1/Heb2). The analysis revealed a significant effect of length by rhyme value [*F*_(3, 193)_ = 83.95, *p* < 0.001] but not of language group [*F*_(1, 336)_ = 1.24, *p* = 0.266] and an interaction effect [*F*_(3, 193)_ = 8.31, *p* < 0.001]. *Post hoc* analysis indicated that in mono-syllabic pairs, Heb1 processed R and NR similarly (*p* = 0.374), whereas Heb2 scored significantly higher (*p* < 0.001) in R than NR. Heb1 scored significantly higher (*p* = 0.014) than Heb2 in NR but not in R (*p* = 0.301). In CVCVC, both Heb1 and Heb2 scored significantly lower in R than NR: Heb1 (*p* < 0.001), Heb2 (*p* < 0.001), with Heb2 scoring significantly higher (*p* = 0.018) in R and significantly lower (*p* < 0.001) in NR than Heb1 (Table 3).

**Table 3:** Means and (SD) of accuracy of rhyming and non-rhyming pairs of all stimulus types by language groups Heb1 vs. Heb2, with Heb2 discerned by L1.

	**Heb1**		**Semitic-L1-Heb2**		**Non-Semitic-L1-Heb2**		**Heb2**	
	**R**	**NR**	**R**	**NR**	**R**	**NR**	**R**	**NR**
CVC	0.91 (0.18)	0.87 (0.23)	0.91^***^ (0.13)	0.49 (0.32)	0.94 (0.08)	0.86 (0.22)	0.94^***^ (0.09)	0.75 (0.30)
Total CVCVC	0.46^***^ (0.24)	0.91 (0.10)	0.43^***^ (0.16)	0.76 (0.14)	0.63^***^ (0.24)	0.84 (0.16)	0.57^***^ (0.24)	0.82 (0.16)
Bi-syllabic pairs								
mVCCVC	0.72${}_{\text{a}}^{*}$ (0.29)	0.86 (0.21)	0.77${}_{\text{a}}^{*}$ (0.22)	0.50 (0.26)	0.74_a_ (0.30)	0.80 (0.18)	0.75_a_ (0.28)	0.71 (0.25)
CVCVC types								
Baseline[–CR,+VM]	0.73${}_{\text{a}}^{*}$ (0.19)	0.85 (0.23)	0.78${}_{\text{a}}^{**}$ (0.12)	0.53 (0.25)	0.75_a_ (0.18)	0.80 (0.21)	0.76_a_ (0.16)	0.72 (0.25)
Baseline[–CR,–VM]	0.62${}_{\text{a},\text{b}}^{***}$ (0.25)	0.98 (0.04)	0.59${}_{\text{a},\text{b}}^{***}$ (0.32)	0.92 (0.11)	0.68${}_{\text{a},\text{c}}^{***}$ (0.30)	0.93 (0.12)	0.65${}_{\text{a},\text{b}}^{***}$ (0.30)	0.92 (0.12)
TCR	0.44${}_{\text{c}}^{***}$ (0.33)	0.92 (0.14)	0.50${}_{\text{b}}^{*}$ (0.28)	0.71 (0.26)	0.66${}_{\text{a},\text{b}}^{***}$ (0.28)	0.92 (0.12)	0.61${}_{\text{b}}^{***}$ (0.29)	0.86 (0.20)
HVM	0.32${}_{\text{c}}^{***}$ (0.35)	0.95 (0.09)	0.22${}_{\text{c}}^{***}$ (0.24)	0.90 (0.15)	0.55${}_{\text{b},\text{c}}^{***}$ (0.38)	0.81 (0.32)	0.45${}_{\text{c}}^{***}$ (0.38)	0.83 (0.28)

Significance in comparisons of R with NR in the same language group and stimulus types are marked by asterisks according to conventional critical *P*-values: *p* < 0.05, *p* < 0.01, and *p* < 0.001.

Means in the same column that do not share the sub-script are significantly different.

Heb1, Hebrew native speakers; Heb2, Hebrew-L2 speakers; HVM, highlighted-vocalic melody; NR, non-rhyming pairs; R, rhyming pairs; TCR, transposed-consonantal root.

The same analysis discerning Heb2 by the participants' L1 (language group (Heb1/nS-Heb2/S-Heb2) × length by rhyme value [CVC-NR/CVC-R/CVCVC-NR/CVCVC-R)] revealed significant effects of length by rhyme value [*F*_(3, 187)_ = 70.41, *p* < 0.001], language group [*F*_(2, 349)_ = 17.81, *p* < 0.001], and an interaction effect [*F*_(6, 187)_ = 7.56, *p* < 0.001]. *Post hoc* analysis indicated that Heb1 scored similarly (*p* = 0.336) in R and NR in CVC but significantly lower (*p* < 0.001) in R than NR in CVCVC. In CVC, S-Heb2 scored significantly lower (*p* < 0.001) in NR than R, while nS-Heb2 approached significance (*p* = 0.053), with higher accuracy in R. No significant differences were shown in R in CVC between Heb1 and nS-Heb2 (*p* = 0.218), Heb1 and S-Heb2 (*p* = 0.864), and nS-Heb2 and S-Heb2 (*p* = 0.483). In CVCVC, both nS-Heb2 (*p* < 0.001) and S-Heb2 (*p* < 0.001) scored significantly lower in R than NR. Interestingly, nS-Heb2 scored significantly higher than S-Heb2 (*p* = 0.006) and Heb1 (*p* < 0.001), but no significant difference was shown between S-Heb2 and Heb1 (*p* = 0.667) (Table 3).

These results could be taken to show that Hebrew-L2 process mono- and bi-syllabic pairs similarly to Hebrew native speakers. However, when discerned by L1, non-Semitic-L1 is similar to Hebrew native speakers in CVC pairs, whereas Semitic-L1 is similar to Hebrew native speakers in CVCVC pairs.

#### 3.1.2. Within the bi-syllabic stimulus types

Since putting all templates in one basket might conceal differences of specific particularities, we sought to examine whether the accuracy rates of Heb2 are similar to those of Heb1 in R and NR within and between the different stimulus types. Table 3 presents a comparison of language group (Heb1/Heb2) by stimulus type by rhyme value (CVC-NR/CVC-R/mVCCVC-NR/mVCCVC-R/TCR-NR/TCR-R/HVM-NR/HVM-R/B(+)-NR/B(+)-R/B(–)-NR/B(–)-R). The analysis revealed a significant effect of stimulus type by rhyme value [*F*_(11, 204)_ = 59.30, *p* < 0.001] but not of language group [*F*_(1, 986)_ = 1.77, *p* = 0.184] and an interaction effect [*F*_(11, 204)_ = 4.82, *p* < 0.001]. *Post hoc* analysis showed that Heb1 scored significantly lower in R than NR in all the bi-syllabic pairs [mVCCVC (*p* = 0.004), TCR (*p* < 0.001), HVM (*p* < 0.001), B(+) (*p* = 0.002), and B(–) (*p* < 0.001)] but not (*p* = 0.374) in the mono-syllabic CVC. Heb2 scored significantly lower in R than NR in the bi-syllabic CVCVC types: TCR (*p* < 0.001), HVM (*p* < 0.001), and B(–) (*p* < 0.001), but similarly in R and NR in stimuli with identical templates in a pair: B(+) (*p* = 0.327) and mVCCVC (*p* = 0.417), and significantly higher (*p* < 0.001) in R than NR in CVC.

The same analysis discerning Heb2 by the participants' L1 (language group (Heb1/nS-Heb2/S-Heb2) × stimulus type [CVC-NR/CVC-R/mVCCVC-NR/mVCCVC-R/TCR-NR/TCR-R/HVM-NR/HVM-R/B(+)-NR/B(+)-R/B(–)-NR/B(–)-R)] revealed a significant effect of stimulus type by rhyme value [*F*_(11, 197)_ = 49.15, *p* < 0.001], language group [*F*_(2, 969)_ = 22.61, *p* < 0.001], and an interaction effect [*F*_(22, 197)_ = 5.93, *p* < 0.001]. *Post hoc* analysis showed that Heb1 scored significantly higher in NR than R in all the bi-syllabic pairs (mVCCVC (*p* = 0.003), TCR (*p* < 0.001), HVM (*p* < 0.001), B(+) (*p* = 0.002), and B(–) [*p* < 0.001)] but not in the mono-syllabic CVC pairs (*p* = 0.336). S-Heb2 showed significant differences in all the bi-syllabic types; however, unlike the Heb1, not always the NR was higher than R: S-Heb2 scored significantly higher in R than NR in CVC (*p* < 0.001), mVCCVC (*p* = 0.002), and B(+) (*p* = 0.001) but significantly lower in R than NR in TCR (*p* = 0.015), HVM (*p* < 0.001), and B(–) (*p* < 0.001). nS-Heb2 also scored significantly lower in R than NR in the TCR (*p* < 0.001), HVM (*p* < 0.001), and B(–) (*p* < 0.001), but no significant differences were shown in mVCCVC (*p* = 0.317) and B(+) (*p* = 0.284), and in CVC, approaching significance (*p* = 0.053) with higher scores in R (Table 3).

These findings indicate that in the three stimulus types, namely TCR, HVM, and B(–), all the participants demonstrated a low accuracy rate in rhyming pairs. In the other three types [CVC, mVCCVC, B(+)], Semitic-L1 showed a low accuracy rate in non-rhyming pairs, while non-Semitic-L1 showed no difference between rhyming and non-rhyming pairs; both Semitic-L1 and non-Semitic-L1 contrast with Hebrew native speakers, whose scores were significantly lower in rhyming pairs.

### 3.2. Rhyming pairs in bi-syllabic stimulus types

#### 3.2.1. Within language groups

Next, we sought to examine the impact each bi-syllabic stimulus type had on each language group. Based on the analysis in the previous section, we compared accuracy in bi-syllabic rhyming pairs between the five stimulus types [mVCCVC, B(+), B(–), TCR, HVM] in each language group (Table 3, letters). For Heb1, accuracy in mVCCVC was significantly higher than TCR (*p* < 0.001) and HVM (*p* < 0.001) but not than B(+) (*p* = 0.855) and B(–) (*p* = 0.060) although approaching significance; significantly lower in B(–) than B(+) (*p* = 0.015); significantly lower in HVM than TCR (*p* = 0.047), with both TCR and HVM significantly lower than B(–) [TCR (*p* = 0.001), HVM (*p* < 0.001)] and B(+) [HVM and TCR (*p* < 0.001)]. For the Heb2, accuracy in mVCCVC was significantly higher than TCR (*p* = 0.017) and HVM (*p* < 0.001) but not than B(+) (*p* = 0.910) and B(–) (*p* = 0.072) although approaching significance, significantly lower in B(–) than B(+) (*p* = 0.024); significantly lower in HVM than TCR (*p* = 0.013), B(+) (*p* < 0.001) and B(–) (*p* = 0.001), and significantly lower in TCR than B(+) (*p* < 0.001) but not B(–) (*p* = 0.490). Breaking down the Hebrew-L2 by the participants L1 showed that the similar trends between Heb1 and Heb2 were due to the Semitic-L1 Hebrew-L2 speakers. S-Heb2 demonstrated a similar trend to Heb1, with accuracy in mVCCVC significantly higher than TCR (*p* < 0.010) and HVM (*p* < 0.001) but not B(+) (*p* = 0.948) and B(–) (*p* = 0.073) although approaching significance; significantly lower in B(–) than B(+) (*p* = 0.027); significantly lower in HVM than B(+) (*p* < 0.001), B(–) (*p* = 0.001), and TCR (*p* = 0.016); and TCR lower than B(+) (*p* = 0.002), but not B(–) (*p* = 0.369). In contrast, nS-Heb2 demonstrated accuracy significant lower in HVM than mVCCVC (*p* = 0.009) and B(+) (*p* = 0.002), without any other differences [mVCCVC–B(+) (*p* = 0.927), mVCCVC–B(–) (*p* = 0.327), mVCCVC–TCR (*p* = 0.240), TCR–HVM (*p* = 0.139), TCR–B(+) (*p* = 0.144), TCR–B(–) (*p* = 0.804), HVM–B(–) (*p* = 0.077), and B(+)–B(–) (*p* = 0.206)].

**Figure 1:**
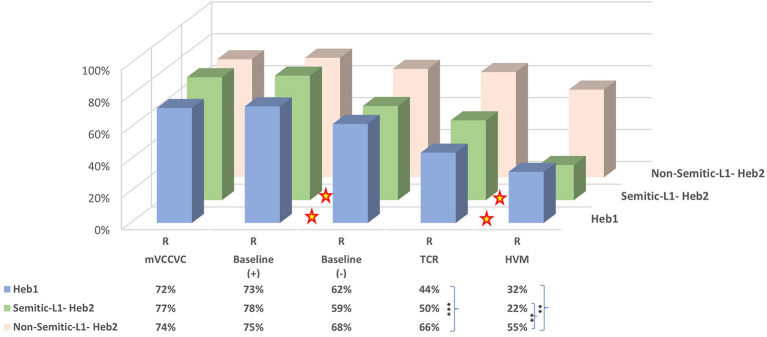
Rhyming bi-syllabic pairs by language groups (distinguished by L1). Colored stars indicate significant differences at *p* < 0.05 between B(–) and B(+) and between TCR and HVM for Heb1 and S-Heb2 language groups. Asterisks in the Data Table indicate significant differences between language groups marked by the curly brackets (in TCR and HVM) according to conventional critical *P*-values: *p < 0.05, **p < 0.01, and ***p < 0.001.

These findings indicate that the Semitic native speakers (Hebrew native speakers and Semitic-L1) show a similar cascade of accuracy mVCCVC=B(+)>B(–)>(Heb1)/ = (S-Heb2)TCR>HVM, which differs from the results of the non-Semitic-L1 whose accuracy is lower only in HVM compared to mVCCVC and B(+). Two major differences between non-Semitic-L1 and Semitic native speakers are the differences shown between B(+) and B(–) and among Semitic native speakers between TCR and HVM, which are not shown among non-Semitic-L1 speakers (Figure 1, colored stars).

#### 3.2.2. Between language groups

Next, we wanted to test whether the accuracy rate in rhyming pairs in the different stimulus types differed between language groups. Comparing the rhyming pairs from the previous analysis of the different bi-syllabic stimulus types between Heb1 and Heb2 indicated no difference between language groups in mVCCVC (*p* = 0.543), B(+) (*p* = 0.385), and B(–) (*p* = 0.525). However, significant differences were shown in the TCR (*p* = 0.004) and approaching significance (*p* = 0.054) in HVM (Figure 1). Breaking down the Heb2 to non-Semitic-L1 and Semitic-L1 speakers showed that this was due to the non-Semitic-L1-Heb2 only: In TCR, nS-Heb2 scored significantly higher than Heb1 (*p* = 0.001) but not than S-Heb2 (*p* =0.085), without a difference between Heb1 and S-Heb2 (*p* = 0.476); and in HVM, nS-Heb2 scored significantly higher than Heb1 (*p* = 0.002) and S-Heb2 (*p* = 0.002), without a difference between Heb1 and S-Heb2 (*p* = 0.321) (Figure 1, asterisks in Data Table).

These findings indicate that both groups of Semitic native speakers (Heb1 and S-Heb2) scored lower than non-Semitic-L1 in the HVM. Interestingly, in TCR, only Hebrew native speakers scored lower than non-Semitic-L1, while Semitic-L1 showed no difference from either non-Semitic-L1 or Hebrew native speakers.

### 3.3. Varying vs. transposed-consonantal roots

Next, we wanted to probe if the lack of difference for nS-Heb2 between B(+) and TCR remains when the vocalic melody is identical in the entire stimuli sample and not only between pairs by removing the potential impact of grammatical information pronounced in the VM and thus better scrutinizing the impact of the phonological co-occurrence restrictions accentuated in the transposed pairs. To this end, we compared pairs sharing the VM *-a-a-*, half with varying consonantal roots (B(+)), and half with transposed-consonantal roots (TCR) in a pair. Table 4 presents a comparison of stimulus type (Transposed/Varying) × language group (Heb1/nS-Heb2/S-Heb2).

**Table 4:** Means and (SD) of varying vs. transposed-consonantal roots in *-a-a-* rhyming pairs.

**Heb1**		**S-Heb2**		**nS-Heb2**		**Heb2**	
**Baseline**	**Transposed**	**Baseline**	**Transposed**	**Baseline**	**Transposed**	**Baseline**	**Transposed**
0.76^***^ (0.23)	0.43_A_ (0.33)	0.78^*^ (0.13)	0.51_a_ (0.32)	0.78 (0.22)	0.70_b_ (0.28)	0.78^***^ (0.19)	0.65_B_ (0.31)

Significance in comparisons of varying with transposed-consonantal roots in the same language group are marked with asterisks according to conventional critical P-values: p < 0.05, p < 0.01, and p < 0.001.

Means in the same row that do not share the sub-script are significantly different.

Lowercase indicates differences between Heb1, non-Semitic-L1-Heb2, and Semitic-L1-Heb2.

Uppercase indicates differences between Heb1 and Heb2.

Heb1, Hebrew native speakers; Heb2, Hebrew-L2 speakers; nS-Heb2, non-Semitic-L1 Hebrew-L2 speakers; S-Heb2; Semitic-L1 Hebrew-L2 speakers.

The analysis revealed a significant effect of stimulus type [*F*_(1, 193)_ = 30.27, *p* < 0.001], language group [*F*_(2, 193)_ = 7.33, *p* = 0.001], and an interaction effect [*F*_(1, 193)_ = 4.94, *p* = 0.008]. *Post hoc* analysis showed significant differences between varying and transposed CRs for Heb1 (*p* < 0.001) and S-Heb2 (*p* = 0.005) but not for nS-Heb2 (*p* = 0.186). In transposed, nS-Heb2 scored significantly higher than S-Heb2 (*p* = 0.042) and Heb1 (*p* < 0.001), with no difference between S-Heb2 and Heb1 (*p* = 0.347). No difference was shown in varying: S-Heb2 vs. nS-Heb2 (*p* = 0.948) and Heb1 (*p* = 0.696), and Heb1 vs. nS-Heb2 (*p* = 0.535).

These findings corroborate that when the consonantal roots are transposed, the Semitic language speakers, but not non-Semitic-L1, exhibit a significantly lower accuracy rate than varying consonantal roots irrespectively of grammatical information conveyed *via* the vocalic melody.

### 3.4. Rhyming HVM pairs

#### 3.4.1. Binyanim relations

To examine whether awareness of the verbal binyanim relations affects the Hebrew-L2 speakers' language mode, we compared the HVM's three types, two of which express violation of syntactical relations (MIX, Pa'al-Pi'el, Pa'al-Pu'al) × language group (Heb1/Heb2). The analysis revealed a significant effect of language group [*F*_(1, 327)_ = 9.81, *p* = 0.002] but not of binyanim relation types [*F*_(2, 222)_ = 0.49, *p* = 0.616] and no interaction effect [*F*_(2, 222)_ = 0.20, *p* = 0.822]. *Post hoc* analysis showed no difference between the three types for both language groups. However, Heb1 scored significantly lower than Heb2 in the MIX type (*p* = 0.016), which does not express binyanim relations, but not in the types that express binyanim relations Pa'al-Pi'el (*p* = 0.157) and Pa'al-Pu'al (*p* = 0.110).

**Figure 2:**
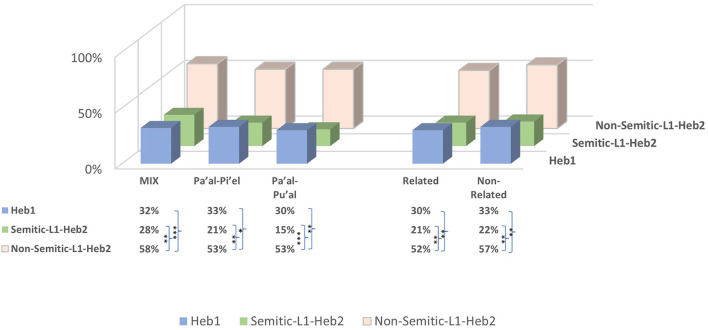
Binyanim relations and semantic relatedness in the HVM rhyming pairs. Asterisks in the data table indicate significant differences between language groups marked by the curly brackets according to conventional critical *P*-values: *p < 0.05, **p < 0.01, and ***p < 0.001.

Analysis discerning Heb2 by the participants' L1 [language group (Heb1/nS-Heb2/S-Heb2) × binyanim relation types (MIX, Pa'al-Pi'el, Pa'al-Pu'al)] revealed a significant effect of language group [*F*_(2, 324)_ = 19.00, *p* < 0.001] but not of binyanim relation types [*F*_(2, 221)_ = 0.77, *p* = 0.463] and no interaction effect [*F*_(4, 221)_ = 0.18, *p* = 0.950]. *Post hoc* analysis showed that in all the HVM types, nS-Heb2 scored significantly higher than Heb1 and S-Heb2: MIX [Heb1 (*p* = 0.001), S-Heb2 (*p* = 0.005)], Pa'al-Pu'al [Heb1 (*p* = 0.003), S-Heb2 (*p* = 0.001)], and Pa'al-Pi'el [Heb1 (*p* = 0.014), S-Heb2 (*p* = 0.007)]. No differences were shown between Heb1 and S-Heb2 for all types: MIX (*p* = 0.661), Pa'al-Pi'el (*p* = 0.289), and Pa'al-Pu'al (*p* = 0.152) (Figure 2, asterisks in Data Table, left).

#### 3.4.2. Semanitc relations

To further examine whether semantic relatedness in the HVM rhyming pairs affects language processing mode between Heb1 and Heb2, we compared language group (Heb1/Heb2) × semantic relatedness (Related/non-Related). The analysis revealed a significant effect of language group [*F*_(1, 220)_ = 7.45, *p* = 0.007] but not of semantic relatedness [*F*_(1, 220)_ = 0.42, *p* = 0.517] and no interaction effect [*F*_(1, 220)_ = 0.00, *p* = 0.995]. *Post hoc* analysis showed that Related and non-Related pairs were similarly processed by Heb1 (*p* = 0.644) and Heb2 (*p* = 0.649). Differences between Heb1 and Heb2 approached significance in both Related (*p* = 0.051) and non-Related (*p* = 0.061) pairs.

Analysis discerning Heb2 by the participants' L1 [language group (Heb1/nS-Heb2/S-Heb2) × semantic relatedness (Related/non-Related)] revealed a significant effect of language group [*F*_(1, 218)_ = 13.83, *p* < 0.001] but not of semantic relatedness [*F*_(1, 218)_ = 0.248, *p* = 0.619] and no interaction effect [*F*_(2, 218)_ = 0.30, *p* = 0.970]. *Post hoc* analysis showed that Related and non-Related pairs were similarly processed by all language groups: Heb1 (*p* = 0.631), S-Heb2 (*p* = 0.959), and nS-Heb2 (*p* = 0.596). By contrast, nS-Heb2 scored significantly higher than both Heb1 and S-Heb2 in both types: Related [Heb1 (*p* = 0.002), S-Heb2 (*p* = 0.003)] and non-Related [Heb1 (*p* = 0.002), S-Heb2 (*p* = 0.002)]. No differences were shown between Heb1 and S-Heb2: Related (*p* = 0.384) and non-Related (*p* = 0.279) (Figure 2, asterisks in Data Table, right).

These findings indicate that when the vocalic melody stands out (due to identical roots), especially but not exclusively, when binyanim relations are involved, and irrespectively of semantic relatedness, both Semitic language speakers exhibit a significantly low accuracy rate than non-Semitic-L1.

### 3.5. Non-stress-matched pairs

To examine the impact of stress on accuracy, we compared language group (Hbe1/Heb2) × non-stress-matched stimulus type [Stress/ Stress(–)]. The analysis showed significant effects of language group [*F*_(1, 219)_ =13.44, *p* < 0.001] and non-stress-matched stimulus type [*F*_(1, 219)_ = 4.64, *p* = 0.032], with no interaction effect [*F*_(1, 219)_ = 0.17, *p* = 0.677]. *Post hoc* analysis showed that accuracy rate was not different between Stress and Stress(–) for Heb1 (*p* = 0.212) and Heb2 (*p* = 0.075). However, Heb1 scored significantly higher than Heb2 in both Stress (*p* = 0.006) and Stress(–) (*p* = 0.018) (Table 5).

**Table 5:** Means and (SD) of non-stress-matched pairs.

	**Heb1**	**Semitic-L1-Heb2**	**Non-Semitic-L1-Heb2**	**Heb2**
Stress	0.77 (0.35) _A_	0.69 (0.23)_ab_	0.54 (39)_b_	0.59 (0.35) _B_
Stress (–)	0.85 (0.30) _A_	0.85 (0.21)_a_	0.64 (0.38)_b_	0.70 (0.35) _B_

Means in the same raw that do not share the sub-script are significantly different.

Lowercase indicates differences between Heb1, non-Semitic-L1-Heb2, and Semitic-L1-Heb2.

Uppercase indicates differences between Heb1 and Heb2.

Heb1, Hebrew native speakers; Heb2, Hebrew-L2 speakers.

The same analysis discerning Heb2 by the participants' L1 (language group (Heb1/nS-Heb2/S-Heb2) × non-stress-matched stimulus type [Stress/ Stress(–)] revealed significant differences in language group [*F*_(2, 216)_ = 9.89, *p* < 0.001] and non-stress-matched stimulus type [*F*_(1, 216)_ = 4.73, *p* = 0.031], with no interaction effect [*F*_(2, 216)_ = 0.179, *p* = 0.836]. *Post hoc* analysis showed no significant differences between the two non-matched-stress types for all language groups: Heb1 (*p* = 0.208), nS-Heb2 (*p* = 0.202), and S-Heb2 (*p* = 0.182). However, Heb1 scored significantly higher than nS-Heb2 in both Stress (*p* = 0.002) and Stress(–) (*p* = 0.002), but not than S-Heb2: Stress (*p* = 0.400) and Stress(–) (*p* = 0.971). S-Heb2 scored significantly higher than nS-Heb2 in Stress(–) (*p* = 0.034) but not in Stress (*p* = 0.182) (Table 5).

These findings indicate that Semitic speakers are susceptible to phonological stress, whether the template is identical or different.

## 4. Discussion

This research explored cross-linguistic influence detached from communicative function by examining activation of language processing mechanisms in an auditory rhyming judgment task, which reflects on the transfer of (meta)linguistic information concerning Hebrew templatic words among Hebrew-L2 adult speakers with Semitic and non-Semitic-L1. The research included comparing Hebrew native speakers with Hebrew-L2 speakers in multi-layer analyses by the bilinguals' L1 due to different L1-L2 similarities and differences and two-resolution levels of transfer: the morphological processing and the sub-lexical morphemes and stress.

The findings show that in the control CVC pairs (not templatic words), accuracy was similarly high in rhyming pairs for all speakers, with no difference compared to non-rhyming pairs for Hebrew native speakers and non-Semitic-L1. Interestingly, Semitic-L1 scored lower in non-rhyming pairs compared to rhyming ones, suggesting an influence of rhyme perception of their mother tongue or insufficient phoneme discrimination in the L2. The results of the CVC pairs show that all language groups activated the linear processing mechanism, as required. The shift to CVCVC showed that all speakers scored lower in rhyming pairs, suggesting morphological processing. However, splitting the CVCVC pairs into stimulus types revealed that Semitic speakers (Hebrew native speakers and Semitic-L1) processed rhyming vs. non-rhyming pairs differently in all stimulus types but not non-Semitic-L1. This research corroborated the need for a multi-layer and high-resolution scrutiny, without which the results balanced out among Hebrew-L2 speakers and within stimulus types, giving an elusive impression of the appliance of the L2 mechanism.

Non-Semitic-L1, like Hebrew native speakers, processed rhyming vs. non-rhyming pairs differently in transposed pairs and in pairs where the vocalic melody is different (B(-) and HVM). However, unlike Hebrew native speakers, when the vocalic melody is identical [mVCCVC and B(+)], non-Semitic-L1 processed rhyming and non-rhyming pairs equally, resembling the processing of non-Hebrew speakers (Laure and Armon-Lotem, 2023b). Thus, in first resolution level, non-Semitic-L1 showed awareness of the morphological processing due to the vocalic melody template. By contrast, in second resolution level, the non-Semitic-L1's high accuracy rate in the HVM sub-type rhyming pairs was different from Semitic speakers, suggesting that lexical-syntactic linguistic information concerning the function of the vocalic melody was not strong enough to transfer. As expected, the phonological linguistic information concerning the root was not transferred. Comparing the varying vs. transposed phoneme rhyming pairs, no difference was shown for non-Semitic-L1 as opposed to Semitic speakers. Furthermore, accuracy was different in the non-stress-matched pairs (non-rhyming pairs) compared to the Hebrew native speakers, suggesting on face value that transfer of linguistic information about the contrastive stress did not occur. However, this language group comprises different L1s, and the results might have balanced, as contrastive stress is language-specific and changes also within language families, e.g., Spanish (contrastive) vs. French (non-contrastive). Together, these findings suggest that non-Semitic-L1 used their L1 processing mechanism with minor activation of L2 due to awareness of the morphological processing.

Semitic-L1 speakers, like Hebrew speakers, processed rhyming vs. non-rhyming pairs differently in transposed pairs and in pairs where the vocalic melody differs (B(-) and HVM). They also processed rhyming vs. non-rhyming pairs differently when the vocalic melody is identical (B(+), mVCCVC), but their results differed from Hebrew native speakers since accuracy was lower in the non-rhyming pairs than rhyming pairs and also differed from non-Semitic-L1, who showed no difference in rhyming vs. non-rhyming processing in these types. In rhyming pairs by stimulus types, Semitic-L1 showed a similar accuracy cascade (mVCCVC=B(+)>B(–)>_(Heb1)_/ =_(S − Heb2)_TCR>HVM) to Hebrew native speakers. Thus, in first resolution level, Semitic-L1 showed awareness of the morphological processing. The results in the second resolution level confirm the awareness of the sub-lexical morphemes: Low accuracy rates were shown in transposed vs. varying consonantal roots and in the HVM, similar to those of Hebrew native speakers. In non-stress-matched pairs, Semitic-L1 also scored similarly to Hebrew native speakers despite the difference in stress between the languages, indicating activation levels of L2 and transfer of linguistic information. The low accuracy in non-rhyming pairs suggests insufficient phoneme discrimination or different rhyme perceptions compared to the Hebrew native speakers. Given that their phoneme discrimination was insufficient and yet their results resembled native speakers when the sub-lexical morphemes are accentuated corroborates their awareness of morphological processing and the sub-lexical morphemes, but was L1 or L2 the governing processing mechanism?

Although both could be equally applied, we find it L1 because of the low accuracy in non-rhyming pairs. The low accuracy could be due to rhyme perception. However, since the results differ from those of Hebrew speakers, it indicates that rhyme perception differs in these two languages, hence the governing mechanism was of L1. Another reason relates to phonological knowledge, which goes with phonetic knowledge expressed in phoneme perception and the ability to discriminate phonemes. Accuracy in non-rhyming pairs in CVC was relatively low, suggesting a non-native-like representation of Hebrew phonemes among the Semitic-L1. Transposed pairs accentuate the root for the phonological co-occurrence restrictions tapping into phonological computational knowledge based on phonemes and their distinguishing features. It is unlikely to activate phonological computational knowledge without native-like phoneme discrimination. Nevertheless, the Semitic-L1 showed accuracy similar to Hebrew native speakers in transposed vs. varying phonemes pairs. Therefore, the awareness of the phonological co-occurrence restrictions without an L2 phonemic representation similar (or close to similar) to Hebrew native speakers is likely to be filtered through L1, as the restrictions are common in Semitic languages.

Taken together, the findings indicate that Hebrew-L2 speakers processed templatic words activating their L1 governing processing mechanism, but not without activation of L2 mechanisms at different awareness levels despite the absence of the need to communicate or comprehend. This is not in accord with the unified competition model's (MacWhinney, 2005) premise that transfer of linguistic information and competition is for communicative function. Moreover, it emphasizes the impact of the non-linguistic parameters (Grosjean, 2001) of form, i.e., the modality, and function, i.e., participating in an experiment, on cross-linguistic influence. Stress is connected to the aural modality, and indeed awareness of L2 contrastive stress was transferred in Semitic-L1. The limitation of this study is its small sample size, which does not allow further investigation of the modality impact by breaking down the non-Semitic-L1 to each of the languages it contains. Furthermore, as we predicted, the awareness level played a role in the transfer. The vocalic melody template, which is linguistic knowledge taught or inducible based on usage, contributed to the awareness of morphological processing in non-Semitic-L1. This agrees with the unified competition model that associates linguistic transfer in adults with the necessity of linguistic awareness.

Interestingly, no activation of L2 was shown concerning the root phonological co-occurrence restrictions. One possible explanation is that, as suggested, this linguistic knowledge is subliminal and none of the non-linguistic parameters triggered its activation. Another explanation draws on the unified competition model, associating entrenchment with the strength of linguistic cues. Further research that includes balanced bilingual children may elucidate this subject. Also, investigation involving formal phonological and phonotactic theories may contribute to the understanding of the cross-linguistic influence concerning the root phonological restrictions in comparison with universal principles, including theories that are not syllable-dependent, like Net Auditory Distance (Dziubalska-Kołaczyk, 2014), that calculate phonemic distance based on their features and markedness.

To conclude, this study expands the scope of cross-linguistic influence research by investigating linguistic arenas when semantics is reduced to better understand how human language is processed. Activation of the L2 language mechanism without semantics projects on the brain plasticity and the neural circuits constructed due to bilingualism. The dynamic activation of all the bilingual languages, without context or semantic demands, enhances the benefit and contribution of bilingualism and cross-linguistic influence on the bilingual's linguistic toolbox. Of importance is the linguistic precision required for obtaining a better understanding of the complexity of the bilingual language mechanisms at work.

## Data availability statement

The raw data supporting the conclusions of this article will be made available by the authors, without undue reservation.

## Ethics statement

The studies involving human participants were reviewed and approved by the Ethics Committee of the Faculty of Humanities, Bar-Ilan University. The patients/participants provided their written informed consent to participate in this study.

## Author contributions

YL: conceptualization, methodology, validation, data collection and curation, formal analysis, investigation, visualization, writing—original draft preparation, and reviewing and editing. SA-L: conceptualization, supervision, funding acquisition, and writing—reviewing and editing. Both authors contributed to the article and approved the submitted version.
