# Supplementary material for: Hebrew-L2 speakers process auditory templatic words through their L1 processing mechanism with awareness of L2
Source: Front Psychol. 2023 Jun 6;14:1164510. doi: 10.3389/fpsyg.2023.1164510 (PMC10281025; doi:10.3389/fpsyg.2023.1164510)
Supplement: Supplementary file 1 [file Table_1.docx]

**Appendix A**

We can extract a participant’s probability of Yes and No by simply counting the number of Yes answered and the number of No answered throughout the test, which is presented in random order, as we assume that a participant does not discriminate on which types (e.g., CVC, TCR, HVM) to choose blindly. A participant that blindly chooses Yes or No has an empirical probability $p_{y}$ and $p_{n}$, respectively. Similarly, the probability of a pair in question being a rhyme is $q_{y}$ and $q_{n}$. This means that the probability of a said participant answering correctly is $p_{n}\cdot q_{n}+p_{y}\cdot q_{y}=p$. We have $m$ questions, and let the number of answers the participant answered correctly be $k.$ With all these parameters, we would like to calculate the probability (or likelihood) of a said participant to answer $k$ correct “random guesses” out of $m$ questions, where each question has a probability $p$ of being answered correctly. We notice that this is a Binomial distribution and is calculated by $\left( \begin{aligned} m \\ k \end{aligned} \right)p^{k}\left( 1-p \right)^{m-k}$. Results indicate that each participant has a probability of less than 0.05 (ranging from 0.051329145 to 2.7312E-48) of achieving their accuracy. Hence, none of the results around 50% indicate by chance accuracy.

**Table A1.** **Participants’ probability achieving their accuracy by chance.**

| **P#** | **Probability** | **P#** | **Probability** | **P#** | **Probability** | **P#** | **Probability** | **P#** | **Probability** |
| --- | --- | --- | --- | --- | --- | --- | --- | --- | --- |
| 1 | 0.00018231 | 64 | 2.6099E-05 | 123 | 3.8973E-19 | 75 | 2.2424E-12 | 157 | 6.3828E-23 |
| 4 | 9.7533E-10 | 76 | 6.0831E-16 | 124 | 1.3565E-17 | 79 | 0.04884712 | 158 | 5.4525E-27 |
| 5 | 7.5764E-10 | 85 | 0.00033073 | 132 | 2.1827E-05 | 83 | 8.4198E-24 | 160 | 0.05132914 |
| 6 | 1.8512E-09 | 104 | 7.8132E-05 | 135 | 4.3354E-06 | 84 | 6.2218E-14 | 161 | 1.3827E-34 |
| 7 | 5.2603E-06 | 106 | 7.7459E-16 | 200 | 0.00297771 | 90 | 0.03699265 | 168 | 1.8887E-27 |
| 9 | 7.5113E-06 | 110 | 0.00233291 | 208 | 0.00010982 | 105 | 1.9029E-11 | 169 | 8.8894E-26 |
| 10 | 1.2846E-21 | 112 | 4.1152E-14 | 213 | 1.2065E-18 | 108 | 2.1599E-13 | 170 | 3.5798E-21 |
| 14 | 2.3474E-08 | 115 | 6.1173E-07 | 235 | 4.3854E-11 | 111 | 6.5407E-05 | 179 | 2.8398E-35 |
| 17 | 6.0557E-06 | 144 | 1.1935E-21 | 236 | 2.352E-11 | 114 | 1.4785E-16 | 181 | 7.699E-22 |
| 19 | 0.00169138 | 151 | 3.9945E-14 | 237 | 6.8635E-13 | 116 | 7.1946E-11 | 182 | 0.03996729 |
| 22 | 4.9279E-39 | 154 | 7.6485E-10 | 238 | 0.00081181 | 127 | 1.8496E-25 | 205 | 1.3365E-17 |
| 29 | 8.1561E-05 | 164 | 2.891E-07 | 241 | 4.0983E-11 | 129 | 2.2451E-10 | 206 | 5.2178E-18 |
| 30 | 0.02611312 | 167 | 7.4477E-17 | 3 | 1.125E-11 | 133 | 8.6369E-16 | 207 | 4.905E-26 |
| 31 | 3.838E-09 | 183 | 8.9265E-14 | 23 | 7.5489E-20 | 134 | 0.00385213 | 211 | 0.00514978 |
| 34 | 6.7853E-34 | 187 | 9.1145E-13 | 26 | 5.5757E-15 | 136 | 6.8116E-07 | 212 | 4.1556E-05 |
| 39 | 1.0961E-27 | 188 | 2.4217E-12 | 32 | 1.7664E-14 | 137 | 2.3488E-16 | 214 | 0.05125535 |
| 42 | 1.2017E-06 | 191 | 1.2171E-10 | 37 | 0.00036225 | 145 | 5.9098E-13 | 218 | 0.01092047 |
| 43 | 2.7312E-48 | 195 | 0.00376875 | 40 | 7.0871E-26 | 146 | 0.00128763 | 219 | 0.0437965 |
| 44 | 7.743E-27 | 196 | 1.0604E-14 | 58 | 1.1156E-14 | 147 | 0.02735561 | 221 | 3.9499E-07 |
| 50 | 8.3543E-18 | 197 | 8.6099E-37 | 59 | 5.3375E-05 | 150 | 4.8759E-07 | 234 | 0.000211 |
| 54 | 2.9296E-10 | 118 | 1.1198E-11 | 65 | 1.3478E-16 | 152 | 3.1602E-25 |  |  |
| 56 | 8.1694E-10 | 120 | 3.0286E-23 | 67 | 1.4326E-08 | 155 | 1.2938E-11 |  |  |
| 61 | 9.0222E-13 | 121 | 5.5549E-19 | 74 | 3.2623E-13 | 156 | 0.00012747 |  |  |

P# = participant number.
